# Stress-Related and Circadian Secretion and Target Tissue Actions of Glucocorticoids: Impact on Health

**DOI:** 10.3389/fendo.2017.00070

**Published:** 2017-04-28

**Authors:** Nicolas C. Nicolaides, Evangelia Charmandari, Tomoshige Kino, George P. Chrousos

**Affiliations:** ^1^Division of Endocrinology, Metabolism and Diabetes, First Department of Pediatrics, National and Kapodistrian University of Athens Medical School, ’Aghia Sophia’ Children’s Hospital, Athens, Greece; ^2^Division of Endocrinology and Metabolism, Center of Clinical, Experimental Surgery and Translational Research, Biomedical Research Foundation of the Academy of Athens, Athens, Greece; ^3^Division of Experimental Genetics, Sidra Medical and Research Center, Doha, Qatar

**Keywords:** stress, stress system, hypothalamic–pituitary–adrenal axis, glucocorticoids, glucocorticoid receptor, circadian endocrine rhythms, clock system

## Abstract

Living organisms are highly complex systems that must maintain a dynamic equilibrium or homeostasis that requires energy to be sustained. Stress is a state in which several extrinsic or intrinsic disturbing stimuli, the stressors, threaten, or are perceived as threatening, homeostasis. To achieve homeostasis against the stressors, organisms have developed a highly sophisticated system, the stress system, which provides neuroendocrine adaptive responses, to restore homeostasis. These responses must be appropriate in terms of size and/or duration; otherwise, they may sustain life but be associated with detrimental effects on numerous physiologic functions of the organism, leading to a state of disease-causing disturbed homeostasis or cacostasis. In addition to facing a broad spectrum of external and/or internal stressors, organisms are subject to recurring environmental changes associated with the rotation of the planet around itself and its revolution around the sun. To adjust their homeostasis and to synchronize their activities to day/night cycles, organisms have developed an evolutionarily conserved biologic system, the “clock” system, which influences several physiologic functions in a circadian fashion. Accumulating evidence suggests that the stress system is intimately related to the circadian clock system, with dysfunction of the former resulting in dysregulation of the latter and *vice versa*. In this review, we describe the functional components of the two systems, we discuss their multilevel interactions, and we present how excessive or prolonged activity of the stress system affects the circadian rhythm of glucocorticoid secretion and target tissue effects.

## The Stress System

The stress system consists of the locus caeruleus/norepinephrine autonomic nervous systems and the hypothalamic–pituitary–adrenal (HPA) axis. These two components interact with each other, as well as with other brain subsystems, such as the mesocortical and the mesolimbic dopaminergic system, which is involved in reward and motivation, the central nucleus of the amygdalae, which generate fear and/or anger, and the arcuate nucleus of the hypothalamus participating in stress system control (1–4). The activity of the stress system is influenced by several neurochemical modulators (e.g., serotonin, acetylcholine, γ-aminobutyric acid, glutamate and endogenous cannabinoids, and benzodiazepines) (1–4). When homeostasis is threatened or perceived by the individual as threatened by stressors, the locus caeruleus/norepinephrine/autonomic nervous systems release norepinephrine in the brain and the systemic circulation, while epinephrine is secreted by the adrenal medulla. On the other hand, the HPA axis is associated with the production and secretion of glucocorticoids by the *zona fasciculata* of the adrenal cortex (1–4). Glucocorticoids play a fundamental role in the maintenance of basal and stress-related homeostasis, regulating many physiologic functions through genomic actions mediated by their cognate intracellular receptor, the glucocorticoid receptor (GR); the latter belongs to the steroid receptor family of the nuclear receptor superfamily of transcription factors (4–8).

The human glucocorticoid receptor (hGR) is encoded by the *NR3C1* gene, which is located in the long arm of chromosome 5 and is composed of 10 exons. The alternative splicing of exon 9 generates the two main protein isoforms of the receptor, the hGRα and the hGRβ. Expressed in every tissue except the suprachiasmatic nucleus (SCN) of the hypothalamus, the hGRα is activated following binding of natural or synthetic glucocorticoids to its ligand-binding domain and binds to the regulatory regions of glucocorticoid-responsive genes through its DNA-binding domain and/or interacts with other transcription factors altering their transcriptional activities (*vide infra*) (6–10). On the other hand, the hGRβ isoform is an enigma in endocrine physiology. Exclusively localized in the nucleus of certain cell types, such as endothelial cells, the hGRβ acts as a dominant-negative inhibitor of hGRα-induced transcriptional activity through well-delineated molecular mechanisms (11–13). Interestingly, this receptor isoform can influence the transcription rate of several genes independently of hGRα (14, 15). Recent studies have demonstrated that hGRβ may be involved in insulin signaling and implicated in gluconeogenesis and inflammation in mouse liver (16, 17). New evidence suggests a pivotal role of the GRβ isoform in the molecular cascades of glioma formation and bladder cancer cells migration (18–20). Further to the alternative splicing of exon 9, Lu and Cidlowski showed that the initiation of the hGRα mRNA translation might occur through eight different sites giving rise to receptor isoforms with variable N-terminal domains: hGRα-A (classic GRα), hGRα-B, hGRα-C1, hGRα-C2, hGRα-C3, hGRα-D1, hGRα-D2, and hGRα-D3, which have distinct properties in terms of intracellular localization and transcriptional activity (10, 21). We assume similar translation processing of the GRβ isoform.

At the cellular level, the glucocorticoid signaling pathway is initiated by ligand-induced activation of the primarily cytoplasmic hGRα, which dissociates from chaperon heat shock proteins and immunophillins, and translocates into the nucleus, where it binds, as homo- or heterodimer, to specific DNA sequences, the glucocorticoid response elements, within the regulatory regions of target genes, thereby influencing their transcription in a positive or negative fashion (4, 6–8, 10). In addition to direct hGRα binding to glucocorticoid-responsive genes, glucocorticoids can influence the transcription of several other genes independently of DNA binding. Indeed, the activated hGRα isoform can interact, possibly as a monomer, with other transcription factors, such as the nuclear factor-κB, the activator protein-1, and the signal transducers and activators of transcription, suppressing or inducing their transcriptional activity (4, 6–8, 10). In addition to the well-described genomic actions, glucocorticoids can induce some cellular effects in a very short-time frame. These effects are referred to as “non-genomic glucocorticoid actions” and are likely to be mediated by membrane-bound GRs, which may trigger the activation of kinase signaling pathways (22–24).

## The Circadian Clock System

To adjust their daily activities to light/dark changes, organisms have developed a highly conserved timekeeping system, the circadian clock system (from the Latin “circa diem” meaning “approximately a day”), which creates internal rhythmicity under the influence of day/night cycles. This regulatory system is composed of a central “master” clock located in the SCN of hypothalamus, and peripheral “slave” clocks, which are ubiquitously expressed in all tissues (25–27). Importantly, peripheral clocks are tightly synchronized to the central clock through as yet unknown possibly neural or neuroendocrine mechanisms. The central clock influences virtually all physiologic functions, such as sleep/wakefulness, feeding, thermoregulation, energy expenditure, glucose homeostasis, and the activity of the HPA axis. Similarly, peripheral clocks regulate several functions of their residing tissues, ultimately contributing to the homeostasis of living organisms (25–28).

**Figure 1:**
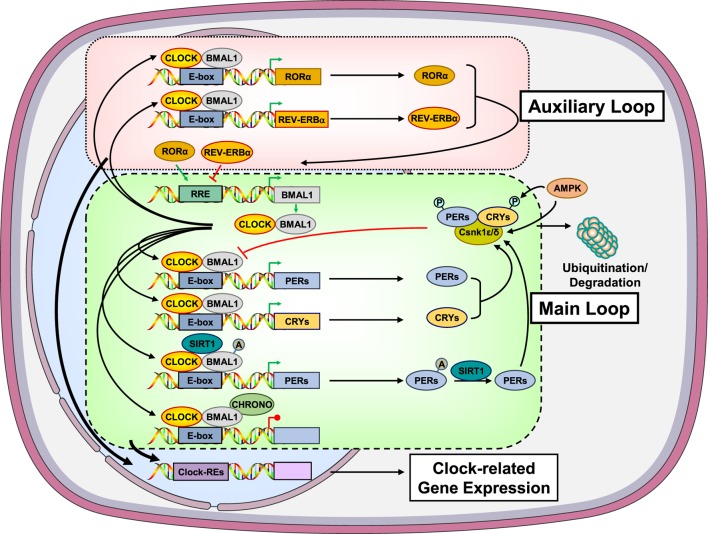
**Molecular components of the main and auxiliary transcriptional/translational loops of the circadian clock system**. In the main transcription loop, the heterodimer CLOCK/BMAL1 causes upregulation of *Pers* and *Crys*. PERs and CRYs undergo phosphorylation by the Csnk1ε/δ and translocate to the nucleus suppressing the transcriptional activity of the CLOCK/BMAL1. Moreover, CLOCK/BMAL1 influences the transcription rate of several other clock-related genes, such as RORα and REV-ERBα, giving rise to an auxiliary transcription loop. AMPK participates in the main transcription loop by phosphorylating CRYs, PERs, and Csnk1ε. SIRT1 functions as a counter-regulatory mechanism for the acetyltransferase activity of the CLOCK by deacetylating BMAL1, PER2, and histone 3. CHRONO, a recently identified BMAL target was found to interact with BMAL1, repressing the main transcription loop *via* recruitment of histone deacetylase 1. A, acetyl residue on the acetylated molecules; AMPK, adenosine monophosphate-activated protein kinase; BMAL1, brain–muscle–arnt-like protein 1; CHRONO, ChIP-derived repressor of network oscillator; CLOCK, circadian locomotor output cycle kaput; CRYs, cryptochromes; Csnk1ε/δ, casein kinase 1ε/δ; P, phosphate residue on the phosphorylated molecules; PERs, periods; RORα, retinoic acid receptor-related orphan nuclear receptor α; SIRT1, sirtuin 1. Modified from Ref. (32).

At the cellular level, the circadian clock system is composed of transcriptional/translational loops, which create an intrinsic, self-oscillating circadian rhythm in both the central and peripheral clocks (Figure 1). At the molecular level, these feedback loops are mediated by the circadian locomotor output cycle kaput/brain–muscle–arnt-like protein 1 (CLOCK/BMAL1) heterodimer and other negative transcription factors, such as the Periods (PER1, PER2, and PER3) and Cryptochromes (CRY1 and CRY2). In the principal or core transcription loop, the activated CLOCK/BMAL1 heterodimer binds to the E-box response elements and induces the expression of *Pers* and *Crys*. These proteins associate with casein kinase (Csnk) 1ε and δ and undergo phosphorylation (29–31). The phosphorylated isoforms then translocate to the nucleus and suppress the transcriptional activity of the CLOCK/BMAL1 heterodimer. In addition, several other clock-related genes, such as retinoic acid receptor-related orphan receptor α (RORα) and reverse viral erythroblastosis oncogene product (REV-ERBα), are upregulated by the CLOCK/BMAL1 heterodimer, forming an auxiliary loop, which stabilizes the transcriptional activity of the core loop. The transcription factors of both principal and auxiliary loops can modulate the expression of many clock-responsive genes in various tissues (27, 29–31).

In addition to light/dark signals, the circadian clock system is strongly influenced by several metabolic inputs, particularly those associated with ingestion of food (Figure 1) (33–39). Indeed, the adenosine monophosphate-activated protein kinase (AMPK), a tissue sensor and master regulator of energy balance, seems to influence the activity of the clock system through energy-dependent signals. AMPK does so by phosphorylating CRYs and PERs leading to their degradation. AMPK can also cause destabilization of PERs indirectly by increasing the activity of Csnk1ε through phosphorylation, resulting in Csnk1ε-mediated degradation of PERs (Figure 1) (33–36). In addition to AMPK, the longevity and metabolism-associated sirtuin 1 (SIRT1) was demonstrated to deacetylate BMAL1, PER2, and histone 3 depending on the NAD+ cellular levels, possibly functioning as a counter-regulatory mechanism for the histone acetyltransferase activity of the CLOCK (Figure 1) (35–39).

## Molecular Interrelations Between the HPA Axis and the Circadian Clock System: The Pivotal Role of Glucocorticoids

### Influence of the Circadian Clock System on the HPA Axis

A growing body of evidence suggests that the stress–responsive HPA axis and the circadian clock system interact with each other at multiple levels (27, 32, 40–45). Indeed, the central clock in the SCN projects neurons in the paraventricular nucleus (PVN) of the hypothalamus providing the basis for the diurnal oscillation of circulating glucocorticoid concentrations, which are higher during the day for diurnal species and at night for nocturnal species (28). In addition, the central clock influences the sensitivity of the adrenal cortex to adrenocorticotropic hormone (ACTH) concentrations through a multisynaptic neuronal pathway (46, 47). On the other hand, in peripheral tissues, the CLOCK/BMAL1 heterodimer represses the hGRα-induced transcriptional activity through CLOCK-mediated acetylation of multiple lysine residues located in the hinge region of the receptor (48–51). In humans, the acetylation status of hGRα is higher in the morning than in the evening, and mirrors the circadian oscillation of cortisol concentrations; therefore, the target tissue glucocorticoid sensitivity reaches a zenith during the evening hours (52). Moreover, CRY1 and CRY2 interact with hGRα leading to reduced DNA binding of the receptor (53).

Recent *in vitro* and *in vivo* studies have identified a novel circadian CLOCK component and BMAL target gene, the *Gm129*, later termed as “*Chrono*” (“ChIP-derived repressor of network oscillator”) (Figure 1) (54, 55). *Chrono* mRNA was found to oscillate in a circadian fashion, which was antiphasic to that of *Bmal1* mRNA, in the mouse SCN, as well as in many peripheral tissues. *Chrono* was showed to encode a 45-kDa protein, called “CHRONO,” which displayed robust circadian oscillation with the opposite phase of BMAL1. CHRONO interacted with BMAL1, CRY2, and DEC2 and functioned as a repressor of the principal transcriptional loop through recruitment of histone deacetylase 1 (55, 56). Moreover, interesting findings from these studies indicated that CHRONO might be a potential link between the circadian clock system and the HPA axis, since this protein interacted with the GR, and *Chrono* knockout mice had increased circulating serum corticosterone concentrations, compared to wild-type mice (56, 57).

### Influence of the Circadian Clock System on Glucocorticoid Secretion

Glucocorticoids, the end products of the HPA axis, play a pivotal role in mediating the stress response and contribute to the tight synchronization of peripheral clocks. These steroid hormones are secreted into the systemic circulation in an ultradian, circadian, and stress-related fashion (58). The ultradian or pulsatile pattern of glucocorticoid release is characterized by a time period of 80–110 min in humans, and its activity is independent of SCN input (59). The circadian release of glucocorticoids is influenced by three factors: (i) the SCN-activated HPA axis, (ii) the SCN-derived autonomic innervation of the adrenal glands through the splanchnic nerve, and (iii) local adrenal clocks (60). As mentioned earlier, SCN neurons send projections into the area of PVN of hypothalamus, thereby creating the diurnal fluctuation of corticotropin-releasing hormone (CRH), arginine vasopressin (AVP), ACTH, and glucocorticoids (28). Furthermore, SCN neurons transmit light information to the adrenal glands *via* splanchnic nerve innervation and increase glucocorticoid release in an HPA-independent fashion (46, 47). The transmission of the light signal to the adrenal cortex is likely to be mediated by catecholamines and/or neuropeptides produced by the adrenal medulla (61). In addition to the photic transmission, the SCN-guided autonomic innervation also alters the sensitivity of the adrenal cortex to ACTH concentrations (46, 47). As already known in the 1960s and as recently confirmed, isolated adrenal gland tissues and cells display robust circadian secretion of steroids, suggesting that the SCN input is not the only prerequisite for diurnal oscillation of the adrenal transcriptome (62). Indeed, adrenal glands harbor their own circadian clocks influencing the rhythmic expression of approximately 10% of the adrenal genome (63). Among genes regulated by adrenal circadian clocks, the steroidogenic acute regulatory protein, a rate-limiting gene encoding a cholesterol transporter into the mitochondria, is rhythmically expressed under the transcriptional control of the adrenal circadian BMAL1 (64).

### Influence of the HPA Axis/Glucocorticoids on the Circadian Clock System

Glucocorticoids, through binding to the hGRα, can effectively reset the activity of peripheral clocks, adding another level of interaction between the HPA axis and the circadian clock system (65). Glucocorticoids alter circadian oscillations of several clock-related genes, including *Pers*, by phase shifting their expression in peripheral organs (66). Therefore, *Per1* and *Per2* genes are upregulated, causing a phase delay of the peripheral clocks, but not the SCN master clock (67). Moreover, the activated hGRα transrepresses the *Rev-ERB*α and *ROR*α genes, influencing directly the activity of the auxiliary transcriptional/translational loop and indirectly that of the main loop (68). At the tissue level, glucocorticoids regulate several local oscillators in different brain areas, as well as in peripheral tissues, as demonstrated by rodent studies. Chronic administration of prednisolone or adrenalectomy strongly influences the expression of clock-related genes [reviewed in Ref. (69)]. In the PVN, adrenalectomy causes a reduction in the amplitude of the expression of *Per1*, whereas acute stress results in the increased expression of the same gene (70, 71). In several limbic areas, many clock genes are suppressed by adrenalectomy, such as the *Per2*. The expression of the latter was shown to be restored when adrenalectomized rats were given corticosterone placed in their drinking water (72). In the hippocampus, the expression of *Per1* was influenced by glucocorticoids (73). In peripheral tissues, glucocorticoids modulate local clocks (74–78). In kidney, adrenalectomy causes phase delay in *Per1* oscillations (74). In white adipose tissue, dexamethasone increases the amplitude of oscillations of clock genes in preadipocytes and attenuates them in differentiated adipocytes (75). In bronchial epithelial cells of the lung, the administration of dexamethasone resulted in upregulation of the *Per1* (76). In cardiac muscle tissue, dexamethasone phase shifts the expression of local clock genes (77). In cornea, adrenalectomy causes a phase delay in *Per1* (74). Finally, the bone local clock can be reset by dexamethasone (78).

### Interplay between Stress and Circadian Clock Systems on the HPA Axis/Glucocorticoids

**Figure 2:**
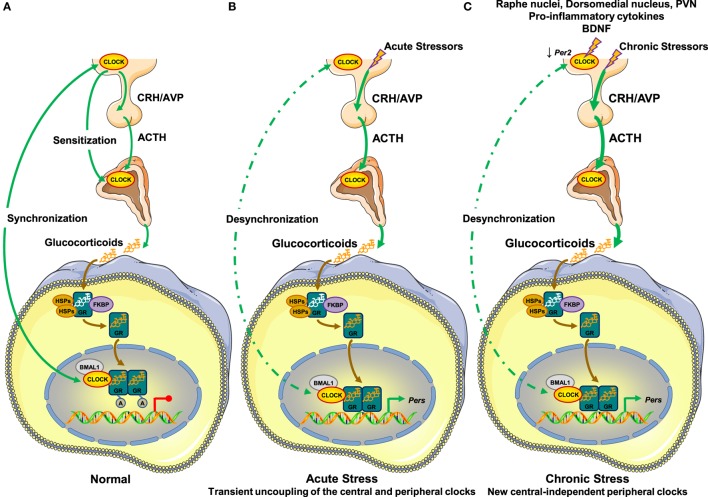
**Molecular interactions between the hypothalamic–pituitary–adrenal (HPA) axis and the circadian clock system**. **(A)** In the absence of stressors, **(B)** under acute stressors, and **(C)** under chronic stressors. **(A)** In normal conditions, the central suprachiasmatic nucleus (SCN) clock creates the diurnal fluctuation of glucocorticoid concentrations by regulating the activity of the HPA axis through neuronal projections and by influencing the adrenal cortex sensitivity to ACTH through the splanchnic nerves. The peripheral clocks are synchronized to the circadian activity of the central clock through unknown mechanisms and suppress the transcriptional activity of the hGRα by CLOCK-mediated acetylation of the receptor. **(B)** Under stressful conditions, acute stressors activate the HPA axis leading to increased glucocorticoid concentrations independently of the central clock-mediated circadian regulation of the HPA axis. In peripheral tissues, glucocorticoids phase shift and reset peripheral clocks leading to uncoupling of the latter from the central clock, granted that the GRα protein is not expressed in the SCN. In addition, the transcriptional activity of the hGRα may be influenced by the phase-shifted peripheral clocks by unknown mechanisms. Following termination of the acute stress, the central clock can reset peripheral clocks to their initial phase. **(C)** In the presence of chronic stressors, the SCN receives indirect glucocorticoid feedback from raphe nuclei, the hypothalamic dorsomedial nucleus, and the paraventricular nucleus. Its activity is also influenced by pro-inflammatory cytokines and BDNF. Chronic stressors trigger the release of glucocorticoids by the adrenal cortex independently of the central clock-mediated diurnal regulation of the HPA axis. This stress-induced glucocorticoid secretion phase shifts and resets peripheral clocks leading to uncoupling of the latter from the central clock. A, acetyl residue on the acetylated molecules; ACTH, adrenocorticotropic hormone; AVP, arginine vasopressin; BDNF, brain-derived neurotrophic factor; BMAL1, brain–muscle–arnt-like protein 1; CLOCK, circadian locomotor output cycle kaput; CRH, corticotropin-releasing hormone; FKBP, FK506-binding protein; GR, glucocorticoid receptor; HSPs, heat shock proteins; PERs, periods.

In the absence of stressors, the central clock regulates the HPA axis activity and influences the sensitivity of the adrenal cortex to ACTH (28, 46, 47). These two mechanisms are responsible for the diurnal fluctuation of circulating glucocorticoid concentrations (28, 46, 47). The peripheral clocks are synchronized to the activity of the central clock through as yet unknown, perhaps neural or endocrine mechanisms (79, 80). In addition, the peripheral clocks suppress the hGRα-induced transcriptional activity through acetylation of the receptor by the CLOCK/BMAL1 heterodimer, possibly functioning as a counter-regulatory mechanism against the circulating glucocorticoid concentrations (48) (Figure 2).

Under stressful conditions, acute stressors induce HPA axis activity, thereby increasing the synthesis and secretion of glucocorticoids by the adrenal cortex. Upon binding to the GRα, secreted glucocorticoids phase shift the expression of several clock-related genes, such as *Per1* and *Per2*, and reset peripheral clocks but not the central clock, granted that GRα is not expressed in SCN neurons (65, 66, 68, 81, 82) (Figure 2). Therefore, the circadian rhythm in peripheral clocks phase shifts from that of central clock under the regulation of the HPA axis, leading to transient uncoupling of the central and peripheral clocks (48). Following termination of the acute stress, the central clock can reset peripheral clocks to their initial phase within a few days (65, 83). hGRα-induced transcriptional activity may be influenced by phase-shifted peripheral clocks in local tissues, but the specific mechanisms remain to be elucidated (Figure 2).

In the presence of chronic or repeated stressors, accumulating evidence suggests that the non-expressing GRα SCN receives indirect glucocorticoid feedback from peripheral tissues expressing GRα, such as the raphe nuclei, the hypothalamic dorsomedial nucleus, and the PVN (84, 85) (Figure 2). Indeed, chronic stressors reduce the expression of *Per2* in the SCN, suggesting an impact of chronic stress on SCN function (86, 87). In addition to glucocorticoids, several other molecules in the periphery (e.g., pro-inflammatory cytokines, brain-derived neurotrophic factor, etc.) provide feedback to the central SCN clock during chronic stress (88, 89) (Figure 2). As chronic or repeated stressors trigger the secretion of glucocorticoids into the systemic circulation independently of the central clock-mediated diurnal regulation of the HPA axis, stress-related glucocorticoid secretion phase shifts and resets peripheral clocks leading to uncoupling of the latter and the central clock (48) (Figure 2). We speculate that a prolonged and/or excessive or deficient adaptive response may not allow the proper rhythmicity of peripheral clocks under the control of the central clock, ultimately leading to several pathologic conditions.

## Clinical Implications

**Figure 3:**
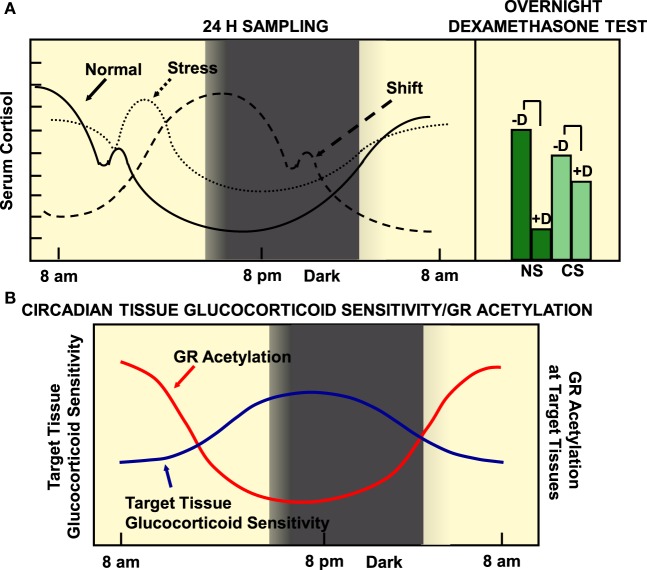
**(A)** Circadian pattern of cortisol secretion in normal humans, CS subjects and rotating shift workers (left panel), and the responses of normal and stressed subjects to overnight dexamethasone suppression test. **(B)** The target tissue sensitivity is lower in the morning and higher at night, mirroring the status of GR acetylation. **(A)** A population of 284 51-year-old men were examined by obtaining a detailed medical history, by performing anthropometry, and by measuring a series of diurnal salivary cortisol concentrations. Participants were asked to fill in a questionnaire about self-perceived stress and underwent a low-dose overnight dexamethasone suppression test. Normal participants were characterized by increased variance, distant zeniths in the morning and nadirs in the evening, and an appropriate suppression in the morning salivary cortisol concentrations following a low-dose dexamethasone suppression test. On the other hand, CS participants showed a decreased variance, evening nadir elevations and morning zenith decreases of cortisol concentrations, as well as an inadequate response to a low-dose dexamethasone suppression test. We speculate that rotating shift workers might be characterized by a phase-delayed curve of salivary cortisol concentrations, compared to that of normal participants. CS, chronically stressed individuals; D, midnight dexamethasone administration; NS, non-stressed individuals. Modified from Ref. (35, 90).

Alterations in circadian release of glucocorticoids have been found in several pathologic conditions, such as mood, metabolic and inflammatory disorders, as well as in cancers (60). On the other hand, chronically stressed (CS) humans, rotating shift workers and subjects frequently exposed to jet lag because of trans-timezone traveling have been demonstrated to be at increased risk for cardiometabolic disorders and their sequelae myocardial infarction and stroke (69). Compared to normal subjects, CS individuals might display an uncoupling between the circadian clock and the HPA axis, a decreased variance between evening nadir and morning zenith cortisol concentrations, as well as an inadequate response to a low-dose dexamethasone suppression test as a result of chronic hyperactivation of PVN CRH and vasopressin secretion (90) (Figure 3). We hypothesize that shift workers or trans-timezone travelers might show a phase-altered curve of cortisol concentrations (Figure 3). Individuals with alterations in circadian secretion of glucocorticoids might develop psychiatric diseases (e.g., anxiety and depression) and autoimmune/inflammatory conditions, with rheumatoid arthritis and asthma as representative examples (91). Furthermore, chronic stress with loss of a proper cortisol circadian rhythm result in glucocorticoid excess-related increased appetite, splachnic obesity, and metabolic disturbances, such as hyperglycemia, insulin resistance, dyslipidemia, osteopenia/osteoporosis, and hypertension (27). All the above pieces of evidence strongly suggest that any dysfunction of the stress system may cause dysregulation of the circadian clock system and *vice versa*.

Several studies in animals and humans have shown that diurnal fluctuations of cortisol concentrations are flattened in obesity. Indeed, genetically obese rats, *db/db* mice, and obese adults display dampened glucocorticoid circadian rhythms (92, 93). In addition, childhood overweight and obesity are associated with a reduction in the amplitude of ultradian glucocorticoid secretion (94). Moreover, subjects carrying specific *Clock* polymorphisms are more susceptible to develop obesity and metabolic syndrome (95, 96). Not only in obesity but also in diabetes mellitus there is a flattened 24-h pattern of cortisol concentrations. In diabetic adults, salivary cortisol was low in the morning and high in the afternoon and evening (97). Finally, mice deficient in the *Cry* gene had a defective suppression of HPA axis, ultimately leading to glucose intolerance and metabolic syndrome (98).

Disruption of circadian rhythms, as often observed in shift workers, may cause mood disorders (99). Mice exposed to a short 7-h light/dark change have higher concentrations of corticosterone and exhibit depressive symptoms (100). On the other hand, subjects suffering from major depression have flattened diurnal glucocorticoid rhythms, probably due to altered secretion of CRH and AVP in the brain (101). They also display defective clock-related gene expression in the peripheral blood cells, as well as in brain tissues (102, 103). Indeed, the rhythmic expression of core clock transcription factor genes, such as *Bmal1, Per1-3, Dec1/2*, and *Rev-erbα* is attenuated in brain regions influencing mood, compared to healthy controls. In addition to depression, several other psychiatric conditions, including bipolar disorder, posttraumatic stress disorder, attention deficit hyperactivity disorder, schizophrenia, and chronic alcoholism, are examples in which the interconnection between the stress system and the circadian clock system is dysregulated (104).

Chronic inflammatory disorders, such as rheumatoid arthritis and asthma, are characterized by worsening symptoms in early morning hours (60). This phenomenon has been attributed to the circadian fluctuation of circulating cytokines, such as interleukin (IL)-1α, IL-6, and tumor necrosis factor-α, which strongly participate in the pathogenesis of these diseases (105, 106). These inflammatory cytokines reach their peak concentrations earlier than cortisol; however, the target tissue glucocorticoid sensitivity is low during that time because of CLOCK-mediated suppressed hGRα-transcriptional and transrepressive activities (48). Furthermore, night-shift workers have increased risk for common infections and multiple sclerosis, indicating that dysregulation of the circadian clock system contributes undoubtedly to the development of inflammatory diseases (107, 108).

Subjects with prolonged night-shift work are also more susceptible to develop several cancers (109). Previous studies have shown that SCN damage, chronic jet lag, as well as *Per2* deficiency contribute to cancer initiation and progression (110–112). On the other hand, patients with breast, ovarian, lung, and kidney cancers have flattened or antiphasic diurnal oscillations of cortisol, compared to normal subjects (60). Future studies are still needed to clarify the molecular mechanisms underlying the association between stress, circadian rhythms, and carcinogenesis.

In addition to metabolic, psychiatric, autoimmune/inflammatory, and malignant disorders, sleep disturbances and disorders have been associated with alterations in circadian secretion of glucocorticoids (69). Indeed, a prolonged or excessive activation of the HPA axis results in insufficient sleep, which, in turn, may cause an elevation of glucocorticoid concentrations and cytokines in the early evening, forming a vicious cycle (69). Moreover, sleep deprivation has been associated with an increase in HPA axis activity in the evening hours, thereby altering the ability of the latter to properly autoregulate itself (113–115). Idiopathic chronic insomnia has been associated with evening hypercortisolism and hypercytokinemia influencing substantially the transcription rate of numerous genes in the brain (116, 117). On the other hand, a dysfunctional HPA axis could promote insomnia (69) or be affected by obstructive sleep apnea (118, 119). Not only chronic insomnia but also chronic fatigue syndrome, fibromyalgia and posttraumatic stress disorder have been linked to consistent alterations in HPA axis activity (120, 121).

## Concluding Remarks and Future Directions: Synthetic Glucocorticoids in the Era of Chronotherapy

Synthetic glucocorticoids have been widely used in the treatment of several inflammatory disorders and hematologic malignancies (122). Since target tissue glucocorticoid sensitivity is lower in the morning and higher in the evening, glucocorticoid analogs should be administered in a time-of-day dependent fashion to achieve a beneficial therapeutic outcome and to avoid their detrimental side effects, such as osteoporosis, weight gain, glucose intolerance, and psychiatric symptoms. Therefore, patients with autoimmune disorders are treated with prednisolone or other synthetic glucocorticoids in the evening, given that the hGRα is less acetylated during that time (106). In addition to inflammatory disorders, chronic administration of glucocorticoids is frequently used as substitution treatment of hypocortisolemic disorders, such as adrenal insufficiency, regardless of its etiology. Approximately two-thirds of hydrocortisone dose is usually given in the morning, while the remainder one-third is administered in two doses (in the mid-day and in the early evening) (123). Recent advances in the therapeutic manipulation of adrenal insufficiency have shown that a dual-release hydrocortisone formulation, which resembles circadian cortisol secretion, results in improved quality of life, decreased body weight and blood pressure, and improved glucose tolerance (124, 125).

Although we have gained important insight in the molecular communication between the stress system and the circadian clock system, there are many physiologic and pathophysiologic aspects of their interrelation that still elude us. The molecular mechanisms underlying resetting of peripheral clocks by glucocorticoids are under intense investigation both in normal and pathologic conditions. Moreover, the tight synchronization of peripheral clocks with the central clock remains poorly understood. Furthermore, our understanding on the function and significance of local adrenal clocks is still increasing. Future *in vitro* and *in vivo* studies will shed light on the functional significance of the cross talk between the stress system and the circadian clock system of living organisms to increase survival chance. Importantly, in parallel with the tremendous progress of molecular, cellular, and structural biology, significant advances in the field of mathematical and computer biosciences will undoubtedly help us have a deeper understanding of system interrelations. Interestingly, accumulating evidence suggests that stress-related “static” signaling pathways can be effectively transformed into functionally predictive computerized kinetic models (126–129). Such efforts will be useful for accurate predictions of a system response to acute or chronic stress, as well as to pharmacotherapy with novel medications.

## Author Contributions

All authors contributed equally to the conception of the work, drafted the manuscript or revisited it critically for important intellectual content, finally approved the version to be published, and agreed to be accountable for all aspects of the work in ensuring that questions related to the accuracy or integrity of any part of the work are appropriately investigated and resolved.

## Conflict of Interest Statement

The authors declare that the research was conducted in the absence of any commercial or financial relationships that could be construed as a potential conflict of interest.
